# Mesotherapy versus Systemic Therapy in the Treatment of Acute Low Back Pain: A Randomized Trial

**DOI:** 10.1155/2011/317183

**Published:** 2010-09-01

**Authors:** Cosimo Costantino, Emilio Marangio, Gabriella Coruzzi

**Affiliations:** ^1^Department of Surgical Sciences, Section of Orthopedy, Traumatology and Functional Rehabilitation, University of Parma, 43121 Parma, Italy; ^2^Department of Clinic Sciences, Section of Respiratory Physiopathology, University of Parma, 43121 Parma, Italy; ^3^Department of Human Anatomy, Pharmacology and Forensic Medicine, University of Parma, 43121 Parma, Italy

## Abstract

Pharmacological therapy of back pain with analgesics and anti-inflammatory drugs is frequently associated with adverse effects, particularly in the elderly. Aim of this study was to compare mesotherapic versus conventional systemic administration of nonsteroidal anti-inflammatory drugs (NSAIDs) and corticosteroids in patients with acute low back pain. Eighty-four patients were randomized to receive anti-inflammatory therapy according to the following protocols: (a) mesotherapy group received the 1st and 4th day 2% lidocaine (1 mL) + ketoprofen 160 mg (1 mL) + methylprednisolone 40 mg (1 mL), then on 7th, 10th, and 13th day, 2% lidocaine (1 mL) + ketoprofen 160 mg (1 mL) + methylprednisolone 20 mg (1 mL) (b) conventional therapy group received ketoprofen 80 mg × 2/die and esomeprazole 20 mg/die orally for 12 days, methylprednisolone 40 mg/die intramuscularly for 4 days, followed by methylprednisolone 20 mg/die for 3 days, and thereafter, methylprednisolone 20 mg/die at alternate days. Pain intensity and functional disability were assessed at baseline (T0), at the end of treatment (T1), and 6 months thereafter (T2) by using visual analogic scale (VAS) and Roland-Morris disability questionnaire (RMDQ). In both groups, VAS and RMDQ values were significantly reduced at the end of drug treatment and after 6 months, in comparison with baseline. No significant differences were found between the two groups. This suggests that mesotherapy may be a valid alternative to conventional therapy in the treatment of acute low back pain with corticosteroids and NSAIDs.

## 1. Introduction

Low back pain affects a high proportion of adult population in the developed countries and has a major impact on health care system and society [1, 2]. Conventional pharmacological therapy to reduce pain, inflammation, and functional disability usually relies upon the extensive use of nonsteroidal anti-inflammatory drugs (NSAIDs), paracetamol (acetaminophen), corticosteroids, and various opioids. However, the major drawback of pharmacological therapy with analgesics and anti-inflammatory drugs is the frequent association with adverse effects [3]; in particular, NSAID-related toxicity is connected to the inhibition of constitutive prostaglandins (PGs), with consequent impairment of gastric mucosal defense and renal homeostasis [4]. On the other hand, the availability of selective cyclooxygenase-2 (COX-2) inhibitors (Coxibs), despite providing a reduction in the gastrointestinal toxicity, resulted in a high risk of developing serious cardiovascular and renal side effects [5, 6]. Chronic therapy with systemic corticosteroids may afford a variety of serious untoward reactions, leading to hypertension, diabetes, glaucoma, gastric ulcer, osteoporosis, and psychiatric disorders [7, 8]. Finally, opioids, used either alone or in combination with paracetamol and/or NSAIDs, may cause a variety of side effects which are dose-limiting and reduce quality of life, bowel dysfunction being one of the most common and persisting problems [9]. Thus, new therapeutic options endowed with comparable efficacy and better safety are warranted. 

Among the various attempts to reduce drug toxicity, the use of local therapy (neural block, intraarticular, or periarticular injections of corticosteroids) has gained popularity among physicians [10, 11], despite some controversies concerning its efficacy as a therapeutic remedy [12].

During the last decades, researchers and patients have become increasingly interested in complementary and alternative medicine (CAM) as a possible mean to ensure efficacy, while improving therapeutic safety [13–15]. Back pain, in particular, is the most common reason for CAM use both in Europe and USA [15]. However, despite the large favour by the general population and several published clinical studies, only few physical treatments are supported by strong scientific evidence [16–18]; likewise, controlled clinical studies evaluating the effectiveness of the most popular CAM therapies used for low back pain are still scarce [19], very few mechanistic studies are available [20, 21], the quality of research is generally poor, and general conclusions are difficult to reach [16].

Mesotherapy was introduced 50 years ago by Michel Pistor, a French physician who utilized this technique as a novel analgesic therapy for a variety of rheumatologic disorders [22]. Mesotherapy is a minimally invasive technique that consists of subcutaneous injections of drugs and, occasionally, plant extracts, homeopathic agents, or other bioactive substances; for this reason, it has been often considered a CAM, rather than a conventional medical therapy [23, 24]. Since its introduction, the use of mesotherapy has been expanded, and therapeutic indications have increased; although most applications are found in osteoarticular pathologies [25–28], over the recent years, this technique has become popular in cosmetic medicine for the treatment of cellulite and fat deposition [29, 30]. 

 Based on these premises, the following study was designed to evaluate the effectiveness of anti-inflammatory drugs (NSAIDs and corticosteroids) administered via mesotherapy in comparison with conventional systemic therapy by oral and intramuscular route, for the treatment of acute low back pain.

## 2. Methods

The study was carried out at the Department of Physical Medicine and Rehabilitation of the University of Parma following the guidelines for experimental investigation with human subjects required by the local University. Informed written consent was obtained from each patient.

### 2.1. Patient Recruitment

Patients were recruited for the study from the Emergency Department between January and May 2007 and checked for eligibility by the clinical investigator. Patients were enrolled into the study, provided that they had been suffering from back pain since no more than 2 weeks and reported a current pain intensity >65 on a 100 mm visual analogic scale (VAS). Exclusion criteria were represented by diabetes, anticoagulant therapy, or pregnancy. Patients were also excluded if they had evidence of cardiovascular, renal, hepatic, gastrointestinal, or psychiatric diseases. Eighty-four patients (44 men, 40 women) aged 24–77 years and suffering from acute low back pain, with cruralgia or sciatalgia were included into the study. Patients could leave the study at any time for any reason.

### 2.2. Study Design

**Figure 1:**
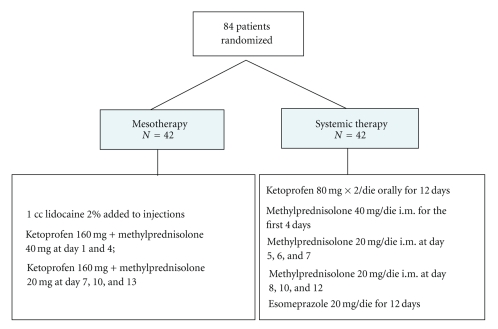
Study design and drug treatment.

Patients who met the eligibility criteria were randomly allocated to receive anti-inflammatory therapy with NSAIDs (ketoprofen) and corticosteroids (methylprednisolone, MP), administered either by mesotherapic technique or by oral/intramuscular route, according to the study plan described in Figure 1.

**Figure 2:**
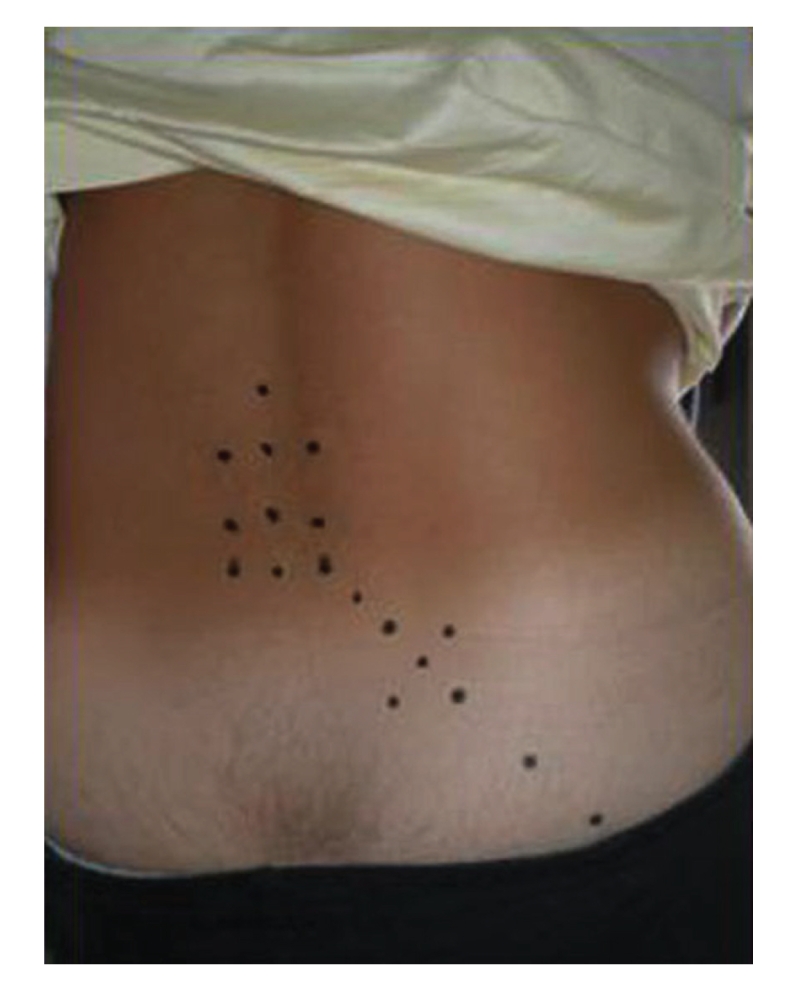
Injection points of a single mesotherapic treatment. Drug injections were administered along the running of sciatic nerve, through specific needles (30 G × 4 mm) (see Methods, for details).

Drug regimen employed in group A (22 men, 20 women) was as follows: 2% lidocaine (1 mL) + ketoprofen 160 mg (1 mL) + MP 40 mg (1 mL) at day 1 and 4, then 2% lidocaine (1 mL) + ketoprofen 160 mg (1 mL) + MP 20 mg (1 mL) at day 7, 10, and 13. Five repeated injections (3 mL for each injection) were administered at inter and paravertebral level along the running of sciatic nerve, through specific needles (30 G × 4 mm), which were inserted deeply for the whole lenght (Figure 2). Lidocaine was used to minimize pain at site of injection.

Group B (22 men, 20 women) received drug therapy according to the following protocol: ketoprofen 80 mg X2/die orally for 12 days; MP intramuscularly 40 mg/die for the first 4 days, then 20 mg/die for 3 days, then 20 mg/die at alternate days. Patients of this group received esomeprazole 20 mg/die for 12 days, as gastroprotective therapy.

### 2.3. Outcome Measures

Self-rated pain intensity was assessed by using the VAS scale (0 = no pain, 100 intolerable pain), a horizontal, unmarked 100 mm scale widely validated to assess pain [31]. Functional disability in the daily life activity was measured by the Roland-Morris disability questionnaire (RMDQ) (varying score from 0 to 24). Both parameters were evaluated at baseline (T0), at the end of the drug treatment (12 days, T1), and at 6 months thereafter (follow up, T2) by two independent observers blind to the pharmacological treatment.

### 2.4. Statistical Analysis

All quantitative data were entered into a specifically designed database (SPSS V 17.01). Chi-Square Mann-Whitney and Kolmogorov-Smirnov test were employed to evaluate the omogeneity of the groups, as for sex or age, respectively. Wilcoxon signed rank test was utilized to analyze the variations among values obtained at baseline (T0), end of treatment (T1), followup (T2), and T0-T1, T1-T2; Krusall-Wallis test was used to analyze differences among T0-T1-T2. *F* test was employed for variance analysis and *T* test for independent data. A *P*  value < .05 was considered statistically significant.

## 3. Results

### 3.1. Patient Characteristics

A total of 84 patients were enrolled into the study. All treated groups were balanced with respect to demographic and baseline characteristics (Table 1). In particular, the patient distribution between the groups was comparable as for sex and age, scores for pain (VAS), and functional disability (RMDQ).

### 3.2. Pain and Functional Disability

**Figure 3:**
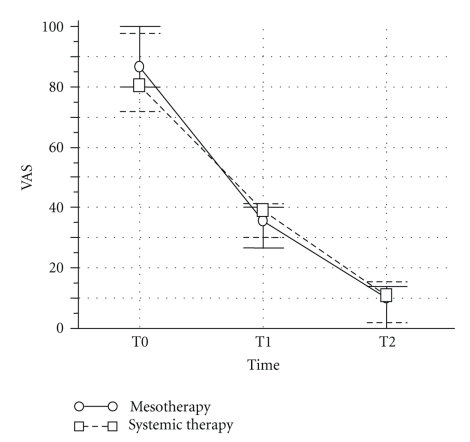
Effect of anti-inflammatory drugs on the reduction of pain, as measured by visual analogic scale (VAS) in patients with acute low back pain. Drug treatment was done either via mesotherapy or via standard systemic route of administration (see methods for details). T0 = baseline, T1 = end of the 12-day treatment and T2 = six months after the end of drug treatment. Values are mean ± SD from 42 patients.

**Figure 4:**
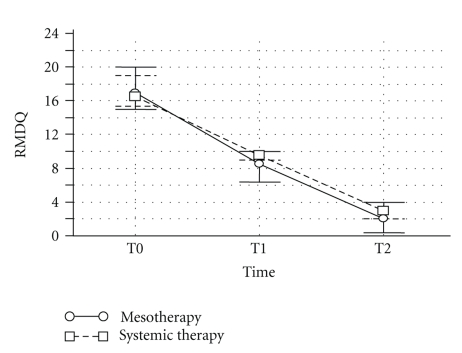
Effect of anti-inflammatory drugs on the reduction of functional disability, as measured by Roland-Morris disability questionnaire (RMDQ), in patients with acute low back pain. Drug treatment was done either via mesotherapy or via standard systemic route of administration. T0 = baseline, T1 = end of 12-day treatment, and T2 = six months after the end of drug treatment. Values are mean ± SD from 42 patients.

In group A (mesotherapy), VAS and RMDQ scores were significantly reduced at the end of the pharmacological treatment (*P*  <  .0001) whereas after 6 months only VAS score was still significantly different from baseline (*P*  =  .04) (Figure 3). In group B (conventional pharmacotherapy), VAS and RMDQ were significantly reduced at the end of the treatment (*P*  <  .0001 and *P*  <  .001, resp.) and both scores were still significantly different from baseline after 6 months (*P*  = .673 and *P*  = .400, resp., versus data at the end of drug administration) (Figure 4). Mesotherapy was well-tolerated and local or allergic reactions were not observed. Minimal pain during and after injection was prevented by the local anaesthetic. Transient bleeding and signs of inflammation occurred in patients at the site of injection, but they resolved in a few days.

## 4. Discussion

The aim of this study was to evaluate the effectiveness of anti-inflammatory drugs administered via mesotherapy in patients with acute low back pain. Present results showed for the first time that the administration of NSAIDs and corticosteroids via mesotherapic technique can provide the same therapeutic benefit as that induced by conventional (oral and intramuscular) drug administration. Indeed, both treatments significantly reduced pain intensity and disability in daily life activity, and the effect was maintained up to 6 months. These results are in accordance with previous studies showing that naproxen and diclofenac, administered via mesotherapy, were more effective than after oral administration [27, 32, 33]. 

**Figure 5:**
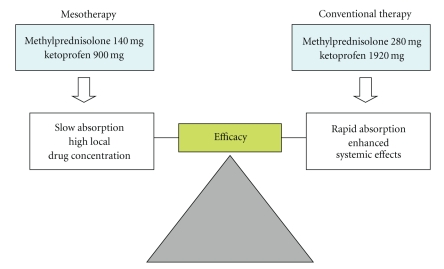
Therapeutic outcome of mesotherapy in comparison with conventional systemic therapy for acute low back pain. These two routes of administration resulted in comparable efficacy, despite the lower (approximately 50%) total amount of drug administered via mesotherapy.

The major finding of this study is the comparable effectiveness of mesotherapy and conventional systemic therapy, despite the lower amount of drugs administered to patients undergoing mesotherapy (41,67% ketoprofen and 50% methylprednisolone) (Figure 5). The comparable efficacy of mesotherapy and conventional therapy, despite different drug dosages, is difficult to explain. Subcutaneous drug administration results in a very slow drug absorption in comparison with other systemic routes, such as oral and intramuscular; thus it could be hypothesized that anti-inflammatory drugs, administered via mesotherapy, achieve a high drug concentration into the subcutaneous tissue and exert local effects in close proximity to inflammatory cells, sensory fibers, and vascular mediators that orchestrate inflammation and pain. 

Although no measurement was made in our study of drug plasma levels after the two routes of administration, it is presumable to hypothesize that mesotherapic treatment resulted in a lower systemic bioavailability of drugs, with consequent lower incidence of adverse reactions. This could offer a great therapeutic advantage, when considering the high rates of adverse effects, associated with NSAID or corticosteroid use in the elderly population [3, 4, 7]. While the use of proton pump inhibitors has significantly limited the incidence of peptic ulceration and other acid-related disorders [34], renal and cardiovascular problems still remain of particular concern. In this connection, both nonselective and COX-2-selective NSAIDs were found to reduce glomerular filtration, increase fluid retention and blood pressure [5, 6], and some highly selective COX-2 inhibitors were found unfavourable in patients with cardiovascular diseases and were withdrawn from the market [5, 35]. Corticosteroids, on the other hand, may have a variety of side effects, including hypertension, diabetes, osteoporosis, glaucoma, and peptic ulcer, which are dose-dependent and related to the systemic drug availability [7, 8]. 

Although mesotherapic techniques used in dermatologic surgery have been associated with a number of adverse effects at sites of injection, including atypical mycobacterial infections [36], urticaria [37], lichenoid drug eruptions [38, 39], and psoriasis [40], no evidence of local reactions were found in the present study. 

In conclusion, results of the study indicate that combined administration of conventional NSAIDs and corticosteroids by mesotherapy is an effective and well-tolerated method for managing low back pain in the short-term, compared with drug therapy administered by oral and intramuscular route. Possible weaknesses of our study are the small number of patients, the short followup period, and the lack of drug plasma level measurements. However, if confirmed in a large trial, these observations could be of potential interest in the pharmacological treatment of low back pain to reduce the adverse effects associated with high plasma levels of antiinflammatory drugs.

## Figures and Tables

**Table 1:** Baseline characteristics of patients.

	Mesotherapy	Conventional systemic therapy	*P*	Test
Males, no.	22	22	.827	*χ* ^2^
Females, no.	20	20	.827	*χ* ^2^
Age, mean (SD), y	53.5 (2.64)	53.0 (2.7)	.895	Kolmogorov-Smirnov
VAS, mean (SD)	86.5 (13.22)	80.5 (5.12)	.554	Mann-Whitney
RMDQ, mean (SD)	17 (13.76)	16.5 (14.56)	.613	Mann-Whitney

VAS indicates visual analogic scale; RMDQ indicates Roland-Morris disability questionnaire; in parenthesis, standard deviation (SD) of the mean.
